# Identification of Selective CYP3A7 and CYP3A4 Substrates and Inhibitors Using a High-Throughput Screening Platform

**DOI:** 10.3389/fphar.2022.899536

**Published:** 2022-07-01

**Authors:** Md Kabir, Elias C. Padilha, Pranav Shah, Ruili Huang, Srilatha Sakamuru, Eric Gonzalez, Lin Ye, Xin Hu, Mark J. Henderson, Menghang Xia, Xin Xu

**Affiliations:** ^1^ Division of Pre-Clinical Innovation, National Center for Translational Sciences (NCATS), National Institutes of Health (NIH), Rockville, MD, United States; ^2^ Department of Pharmacology, The Graduate School of Biomedical Sciences, Icahn School of Medicine at Mount Sinai, New York, NY, United States; ^3^ Novartis Institutes for BioMedical Research, Cambridge, MA, United States

**Keywords:** cytochrome P450, CYP3A7, CYP3A4, drug metabolism, qHTS data analysis, neonates, substrates, inhibitors

## Abstract

Cytochrome P450 (CYP) 3A7 is one of the major xenobiotic metabolizing enzymes in human embryonic, fetal, and newborn liver. CYP3A7 expression has also been observed in a subset of the adult population, including pregnant women, as well as in various cancer patients. The characterization of CYP3A7 is not as extensive as other CYPs, and health authorities have yet to provide guidance towards DDI assessment. To identify potential CYP3A7-specific molecules, we used a P450-Glo CYP3A7 enzyme assay to screen a library of ∼5,000 compounds, including FDA-approved drugs and drug-like molecules, and compared these screening data with that from a P450-Glo CYP3A4 assay. Additionally, a subset of 1,000 randomly selected compounds were tested in a metabolic stability assay. By combining the data from the qHTS P450-Glo and metabolic stability assays, we identified several chemical features important for CYP3A7 selectivity. Halometasone was chosen for further evaluation as a potential CYP3A7-selective inhibitor using molecular docking. From the metabolic stability assay, we identified twenty-two CYP3A7-selective substrates over CYP3A4 in supersome setting. Our data shows that CYP3A7 has ligand promiscuity, much like CYP3A4. Furthermore, we have established a large, high-quality dataset that can be used in predictive modeling for future drug metabolism and interaction studies.

## Introduction

Hepatic drug metabolism plays a key role in drug elimination and overall drug disposition (Di et al., 2006). Cytochrome P450 (CYP) 3A4, belonging to the CYP3A enzyme subfamily, is one of the most abundant and important xenobiotic metabolizing enzymes in adults (Guengerich, 1999). The CYP3A subfamily also includes CYP3A5, CYP3A7, and CYP3A43 (Stevens et al., 2003). CYP3A7 is the major CYP3A enzyme in human fetal, newborn, and infant liver from birth to 1 year old, with a subsequent switch to CYP3A4 after the first year (Stevens et al., 2003; Leeder et al., 2005; Zane et al., 2018; Li and Lampe, 2019). CYP3A7 expression can also be found in a subset of the adult population who carry the CYP3A7*1C allele (Sim et al., 2005), which is associated with lower levels of dehydroepiandrosterone sulfate (DHEAS) in men (Smit et al., 2005) and is linked to mortality in breast and lung cancers (Johnson et al., 2016). Additionally, pregnant women express CYP3A7 in endometrium and placenta, which is believed to protect the fetus from the toxic effects of endogenous steroids (Schuetz et al., 1993). Increased CYP3A7 expression was also observed in primary ovarian cancer and metastasis when compared to normal tissue (Downie et al., 2005).

While CYP3A7 shares 88% homology with CYP3A4, the two enzymes exhibit different functionality (Woodland et al., 2008). Testosterone is an established substrate for the CYP3A family, however CYP3A4 and CYP3A7 generate different primary metabolites, producing 6β-hydroxytestosterone and 2α-hydroxytestosterone, respectively (Kandel et al., 2017; Zane et al., 2018). The difference in regioselectivity between CYP3A4 and CYP3A7 was previously shown with midazolam, using adult and fetal microsomes which are enriched with only one of the enzymes (Gorski et al., 1994). Ohmori et al. (1998) further demonstrated that the 16α-hydroxylase activity of dehydroepiandrosterone (DHEA) and DHEAS was more prevalent in CYP3A7 than CYP3A4. Williams et al. (2002) showed that CYP3A7 had significantly reduced catalytic activity compared to CYP3A4 by monitoring biotransformation of several well-known CYP3A substrates. An additional investigation into the metabolism of glyburide revealed the stereoselectivity of CYP3A7 using human fetal liver microsomes and recombinant CYP3A7 (Schuetz et al., 1993). It has been recently shown that some of the azoles that inhibit CYP3A4 also inhibit CYP3A7, but to a lesser extent (Godamudunage et al., 2018). Collectively, these findings exemplify the distinction between the catalytic functions of the two enzymes. However, the FDA has yet to provide guidance regarding the CYP3A7-specific substrates or inhibitors to be used for *in vitro* drug development and interaction studies (FDA, 2020).

Understanding CYP3A7-mediated metabolism can aid in designing novel drugs specifically targeted for neonates and infants. Particularly with the advancement for newborn screening for rare diseases, such as glutaric aciduria type 1 and homocystinuria, early intervention may help in long-term health outcome. Currently, about 90% of drugs used for neonates and 70% of drugs used in the neonatal intensive care unit (NICU) are administered off-label (Hsieh et al., 2014; Laughon et al., 2014; Avant et al., 2017). Evaluating drug metabolism and drug-drug interactions with a focus on CYP3A7 activity may provide crucial pre-clinical data to avoid adverse drug reaction in neonates (Kumar et al., 2008). Understanding CYP3A7-related metabolism may also aid in developing more effective cancer therapeutics where elevated CYP3A7 expression is implicated. Therefore, we performed a quantitative high-throughput screen with ∼5,000 compounds, including FDA approved and drug-like molecules, to identify potential CYP3A7-selective substrates and inhibitors, compared with CYP3A4. This dataset also serves as a foundation to develop in-silico tools that predict CYP3A7 metabolism and/or inhibition, which will facilitate early drug discovery research for neonates and cancer patients.

## Materials and Methods

### Materials

Potassium phosphate monobasic, potassium phosphate dibasic, albendazole, and dimethyl sulfoxide (DMSO) were purchased from Sigma-Aldrich (St. Louis, MO, United States). Acetonitrile, Optima™ LC/MS grade was purchased from Thermo Fisher (Waltham, MA, United States). Supersome™ containing human CYP3A4 (1 nmol/ml), oxidoreductase, and cytochrome b_5_ (product # 456202; lots 5070002, 6161001, and 9037004), supersome™ containing human CYP3A7 (1 nmol/ml), oxidoreductase, and cytochrome b_5_ (product # 456237; lots 732001, 8045003, 8107003, 8249002, and 9218002), NADPH Regenerating System, Solution A (product # 451220), and Solution B (product # 451200), and low profile Axygen^®^ single well reagent reservoir with 384-bottom troughs were purchased from Corning Inc. (Corning, NY, United States). MCA 96 nested 200 μl (product # 30038619) and 50 μl (product # 30038609) disposable tips were purchased from Tecan (Männedorf, Switzerland). 384-Well polypropylene sample collection plate of 250 µl (product # 186002632), and 100 µl (product # 186002631) were purchased from Waters (Milford, MA, United States).

### Compound Library

The compound library consisted of 2,800 drugs from the National Center for Advancing Translational Sciences (NCATS) Pharmaceutical Collections (Huang et al., 2011a). The additional 2,200 compounds were selected from the NCATS annotated bioactive collection based on their drug-like properties (Gonzalez et al., 2021).

### P450-Glo CYP3A7 and CYP3A4 Enzyme Assays

To identify compounds that inhibit CYP3A7 and/or CYP3A4, P450-Glo assays with luciferin-BE (Luc-BE) and -PPXE (Luc-PPXE) substrates were used to perform quantitative high-throughput screens (qHTS). Different luciferin substrates were used for CYP3A7 and 3A4 assays because 3A4 has multi-substrate sites (Kenworthy et al., 1999). Luc-BE substrate shown the unexpected phenomenon of positive heterotropic cooperativity in CYP3A4 assay (Niwa et al., 2008), therefore Luc-PPXE was found to be more appropriate for use in the CYP3A4 assay. We performed the P450-Glo CYP3A7 assay using Luc-BE as substrate according to the vendor’s recommendation, however, we have not found heterotropic cooperativity to be an issue against this enzyme. Of note, some CYP3A7 selective inhibitors, such as halometasone (see in results section), showed inhibitory effect exclusively when using Luc-BE substrate.

The CYP450 inhibition (P450-Glo™) assay was performed as described previously (Veith et al., 2009; Gonzalez et al., 2021). Reaction mixtures were combined and incubated in medium binding white solid 1,536-well plates (Greiner Bio-One North America Inc., Monroe, NC) using a Flying Reagent Dispenser (FRD, Aurora Discovery, San Diego, CA). Briefly, 2 µl of 40 nM CYP3A7 enzyme-150 µM Luc-BE probe substrate mixture (Promega Corporation, Madison, WI) was pre-incubated with control and test compounds at room temperature for 10 min. The CYP reaction was initiated with the addition of 2 µl of NADPH regenerating system (1.3 mM NADP+, 3.3 mM glucose-6-phosphate, 3.3 mM MgCl_2_, and 0.4 U/ml glucose-6-phosphate dehydrogenase) in 200 mM potassium phosphate buffer, resulting in a final reaction volume of ∼4 µl with 20 nM (4 nM of CYP450 content) of CYP3A7 supersomes. Compounds were tested in multiple concentrations ranging from 3.5 nM to 58 μM, while maintaining the final DMSO concentration <0.6%. The reaction mixture was incubated at room temperature for 1 h. The CYP reaction was stopped with the addition of 4 µl of the detection reagent. The luminescence intensity was quantified after 20 min incubation at room temperature using ViewLux plate reader (PerkinElmer, Shelton, CT). Data were expressed as relative luminescence units. For the P450-Glo CYP3A7 assay, a same protocol was used except the substrate with 25 µM Luc-PPXE (Promega Corporation, Madison, WI). The final supersomal concentration was 10 nM (2 nM of CYP450 content).

### Data Analysis for P450-Glo CYP3A7 and CYP3A4 Assays

Data generated from the CYP3A7 and CYP3A4 assays were analyzed as described previously (Huang et al., 2014; Huang et al., 2016). Raw plate reads were normalized to the positive control 28 µM Ketoconazole (used as 100% inhibition) and DMSO-only (0% inhibition) wells according to the following equation: % Activity = [(V_compound_-V_DMSO_)/(V_DMSO_-V_pos_)] x 100, where V_compound_ denotes the test compound, V_pos_ denotes the median value of the positive control, and V_DMSO_ denotes the median values of the DMSO-only controls. The DMSO-only wells at the beginning and end of the compound plate stack were used to correct the data set by applying a NCATS in-house correction algorithm (Wang et al., 2016). The half-maximum inhibitory concentration (IC_50_) and maximum response (efficacy) value for each compound were obtained by fitting the concentration-response curve to a four-parameter Hill equation (Wang et al., 2010). Compounds were designated as class 1–4 according to the type of concentration–response curve observed. Compounds with class 1.1, 1.2, and 2.1 curves or 2.2 curves with >50% efficacy were considered active (hits); class 4 compounds were inactive; and the other curve classes were considered inconclusive (Inglese et al., 2006; Huang et al., 2011b). CYP3A7-selective hits were identified as ligands that presented potency ≤1 μM, efficacy ≥65% and a ≥10-fold IC_50_ difference with CYP3A4 and vice versa for CYP3A4-selectivity criteria.

The 5,000 compounds were grouped into 508 clusters based on structural similarity (1024-bit CDK fingerprints generated using KNIME (Berthold et al., 2007) with the self-organizing map algorithm (Kohonen, 2006). Each cluster was evaluated for its enrichment of active inhibitors and significance of enrichment as determined by *p*-values from the Fisher’s exact test.

The physiochemical properties of these 5K compounds were evaluated using KNIME Analytics Platform (version 3.7). A One way Anova followed by Tuckey test was performed to determine the statistical significance between common CYP3A4/CYP3A7 hits, CYP3A4-and CYP3A7-selective hits.

### High-Throughput Metabolic Stability Assay and Sample Analysis

Metabolic stability was determined using a previously described method (Di et al., 2006; Shah et al., 2016). Briefly, 1 µM of test compound was combined with 3 pmol of either CYP3A7 or CYP3A4 supersomes in 100 mM potassium phosphate buffer, in a 384-well plate, and incubated at 37°C, under gentle shaking for 20 min. The reaction was initiated with the addition of NADPH regenerating system yielding a total reaction volume of 110 µl. Samples were aliquoted and quenched with acetonitrile at 0, 5, 10, 15, 30, and 60 min. The samples were then centrifuged for 20 min at 3,000 rpm, 6°C and supernatants were extracted for UPLC/MS analysis.

Sample analysis was performed with slight deviation from methods described previously (Di et al., 2006; Shah et al., 2016). Briefly, sample quantification was performed using a Thermo Scientific UltiMate 3,000 Ultra Performance Liquid Chromatography (UPLC) system coupled with a Thermo Scientific QExactive™ High Resolution Mass Spectrometer. Chromatography was resolved on a Waters Acquity UPLC BEH C18 column (1.7 μm, 2.1 × 50 mm), which was heated to 60°C. A 0.7 ml/min mobile phase comprised of A (99.9% water and 0.1% formic acid) and B (99.9% CH_3_CN and 0.1% formic acid) was employed per the following gradient: 0–0.2 min 5% B, 0.2–1.7 min linear gradient from 5 to 95% B, 1.7–2.1 min 95% B. Electrospray ionization was used in positive mode with the detector set at full-scan, ranging from 50 to 1,000 m/z at a resolution of 35,000. TraceFinder 4.2 was used to process the data and integrate the parent compound ion peaks, from which half-life (t_1/2_) and intrinsic clearance (CL_int_) parameters were calculated using IQC-validator, an NCATS database to manage *in vitro* clearance data (Shah et al., 2016).

For classification purposes, a compound was considered CYP3A7-specific substrates if it presents a half-life ≤30 min in CYP3A7 supersomes and >120 min in CYP3A4 supersomes, and vice versa for CYP3A4-specific substrates.

### Docking of Halometasone to CYP3A7 and CYP3A4

The three-dimensional structures of CYP3A7 and CYP3A4 were obtained from the Protein Data Bank (PDB code 7MK8 and 5TE8). The structure of CYP3A4 is complexed with midazolam at the active site (Sevrioukova and Poulos, 2017), while CYP3A7 binds with ligand dithiothreitol (Sevrioukova, 2021). Prior to molecular modeling and docking, the protein structures were prepared and energy-minimized using the Protein Preparation module in the MOE program. Missing residues were added to the structure and mutated residues found in CYP3A7 (R69G/C77G/K244E/K421A/K422A/K424A) were re-modeled to wildtype. The co-factor heme was retained in docking while solvent and small molecule ligands were removed. Docking of halometasone to the active site of CYP3A4 and CYP3A7 were performed using the MOE_Dock. The ligand induced fit protocol was used and the binding affinity was evaluated using the GBVI/WSA score. The top 30 poses from each docking were retained for binding mode analysis. Finally, the optimal binding models of halometasone with CYP3A4 and CYP3A7 were refined using a protocol with stepwise energy minimization and MD simulations in the MOE program.

## Results

### Quantitative High-Throughput Screening P450-Glo Assays

**FIGURE 1:**
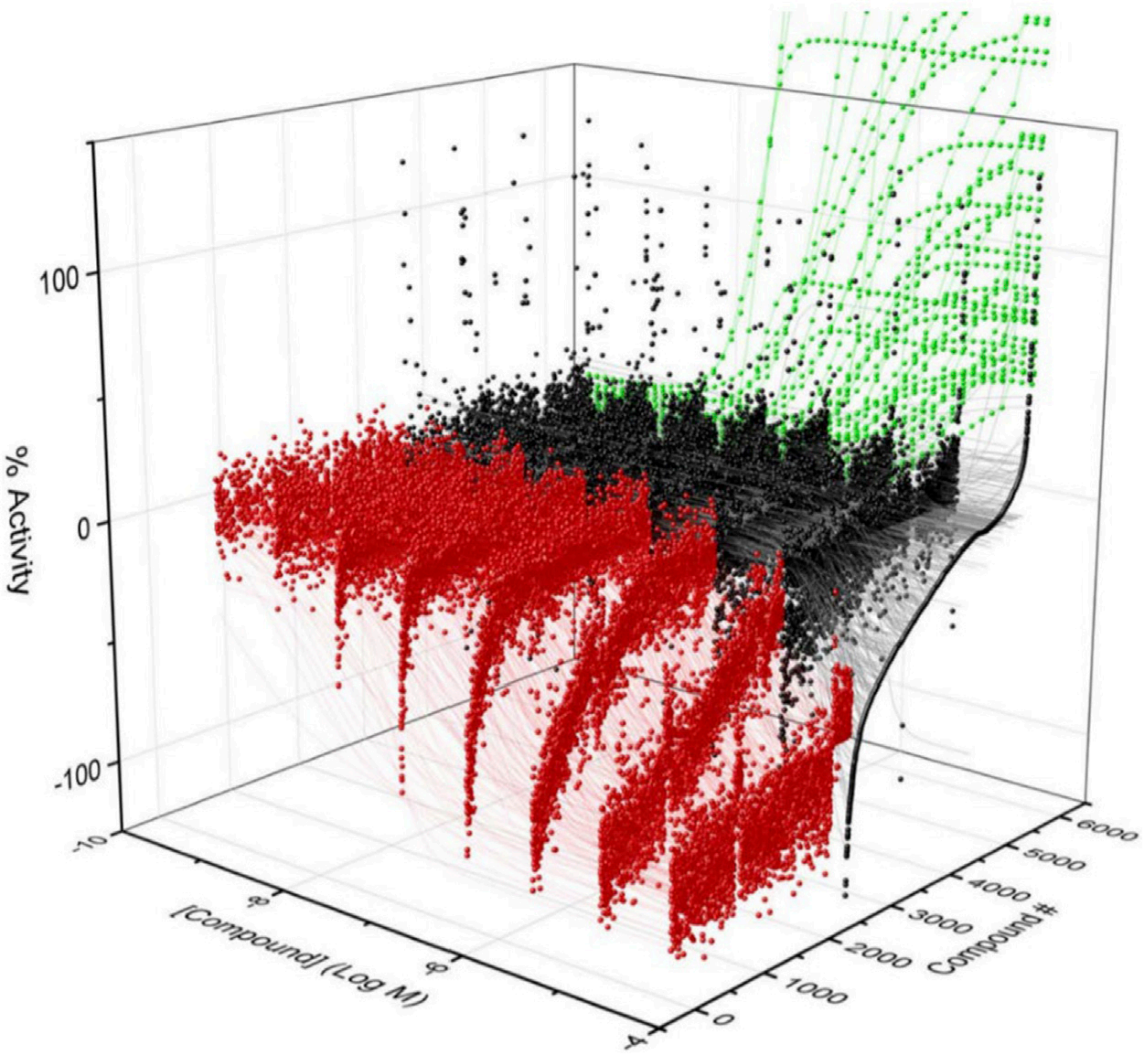
Activity of CYP3A7 in the presence of ∼5,000 test compounds at various concentrations. Red, black, and green represent inhibitors, inactives, and activators, respectively.

**FIGURE 2:**
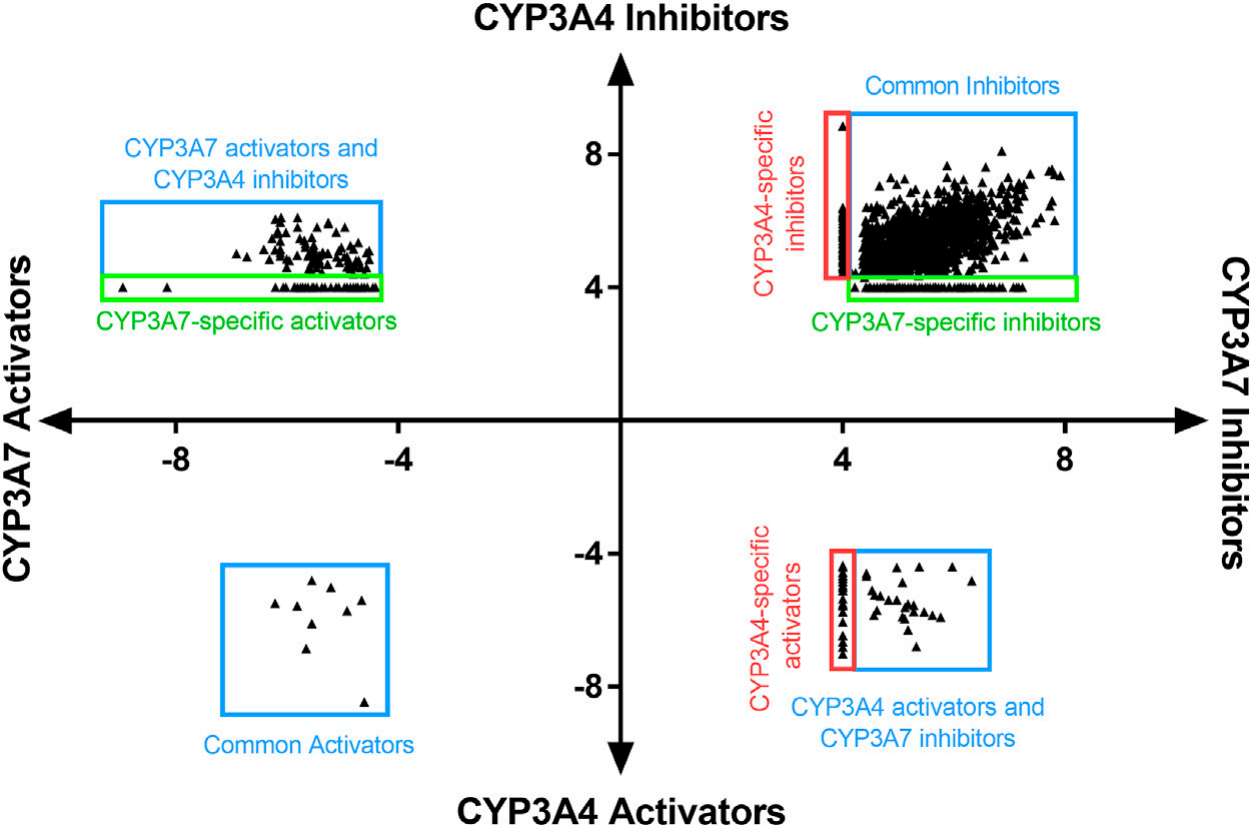
LogIC_50_ of CYP3A7 and CYP3A4 hits. Inhibitors are shown as positive logIC_50_ and activators are shown as negative logIC_50_. Inactive compounds are shown as logIC_50_ of 4 (100 μM, virtually inactive). Bottom left quadrant hits showed negative IC_50_ for both CYP3A4 and CYP3A7 and were categorized as common activators. Top right quadrant shows common inhibitors in the qHTS Glo assay. Top left and bottom right are compounds that shown activation against one enzyme and inhibition against the other. Boundary boxes (red or green boxes) show enzyme specific observations (inhibitor or activator-specific behavior). If IC_50_ was not be able to determined for any enzyme, compounds were excluded from the plot.

To identify compounds that interact with CYP3A7 and/or CYP3A4, a P450-Glo assay was used to perform a qHTS in a 1,536-well plate format. The plate metrics of the qHTS, such as signal-to-background ratio (S/B), coefficient of variation (CV) and Z′ factor were calculated as described previously (Zhang et al., 1999; Huang et al., 2011b) to assess the assay performance. Assays with S/B ≥ 3.0, CV ≤ 10.0, and Z′ factor ≥0.5 are generally considered as good quality. From the current study, the mean ± SD (*n* = 38) of S/B, CV, and Z’ factor are 29.6 ± 4.0, 8.89 ± 3.8, and 0.72 ± 0.1 for CYP3A7, and 35.5 ± 4.3, 14.6 ± 3.4, and 0.56 ± 0.1 for CYP3A4 respectively, suggesting the assays performed well. The IC_50_ of 5,000 compounds was investigated at multiple concentrations ranging from 3.5 nM to 58 μM. A waterfall plot showing the dispersion of the curves of 5,000 compounds divided into inhibitors, inactives, and activators against CYP3A7 is shown in Figure 1. From the primary screening, 2,216 hits were found active for both enzymes (inhibitors or activators); 782 hits were CYP3A7-selective, and 573 hits were CYP3A4-selective. The logIC_50_ of CYP3A7 was plotted against the logIC_50_ of CYP3A4 in Figure 2. The negative logIC_50_ denotes activator behavior of compounds and positive IC_50_ was used for inhibitor behavior in the P450-Glo assay. The logIC_50_ value of 4 means IC_50_ was above 50 μM, the assay top concentration, therefore, the compound was considered inactive in the P450-Glo assay. Most of the compounds were shown to be common inhibitors of CYP3A4 and CYP3A7 (blue boundary box in top right quadrant). The red and green boundary boxes in top right quadrant show compounds that were specific CYP3A4 or CYP3A7, respectively, since compounds inhibited one enzyme while were inactive against the other enzyme (logIC_50_ of 4). Compounds in top left quadrant are CYP3A7 activators and CYP3A4 inhibitors; the green boundary box show compounds where CYP3A4 was inactive but showed CYP3A7 activation. Analogously, in bottom right quadrant, CYP3A4 specific activators and compounds that were found to be CYP3A7 inhibitors and CYP3A4 activators can be found. The bottom left quadrant shows compounds that were common activators of both CYP enzymes. The raw data for this Figure can be found at Supplementary Material S1.

### Identification of CYP3A7 and CYP3A4 Selective Substructures From Quantitative High Throughput Screening

**FIGURE 3:**
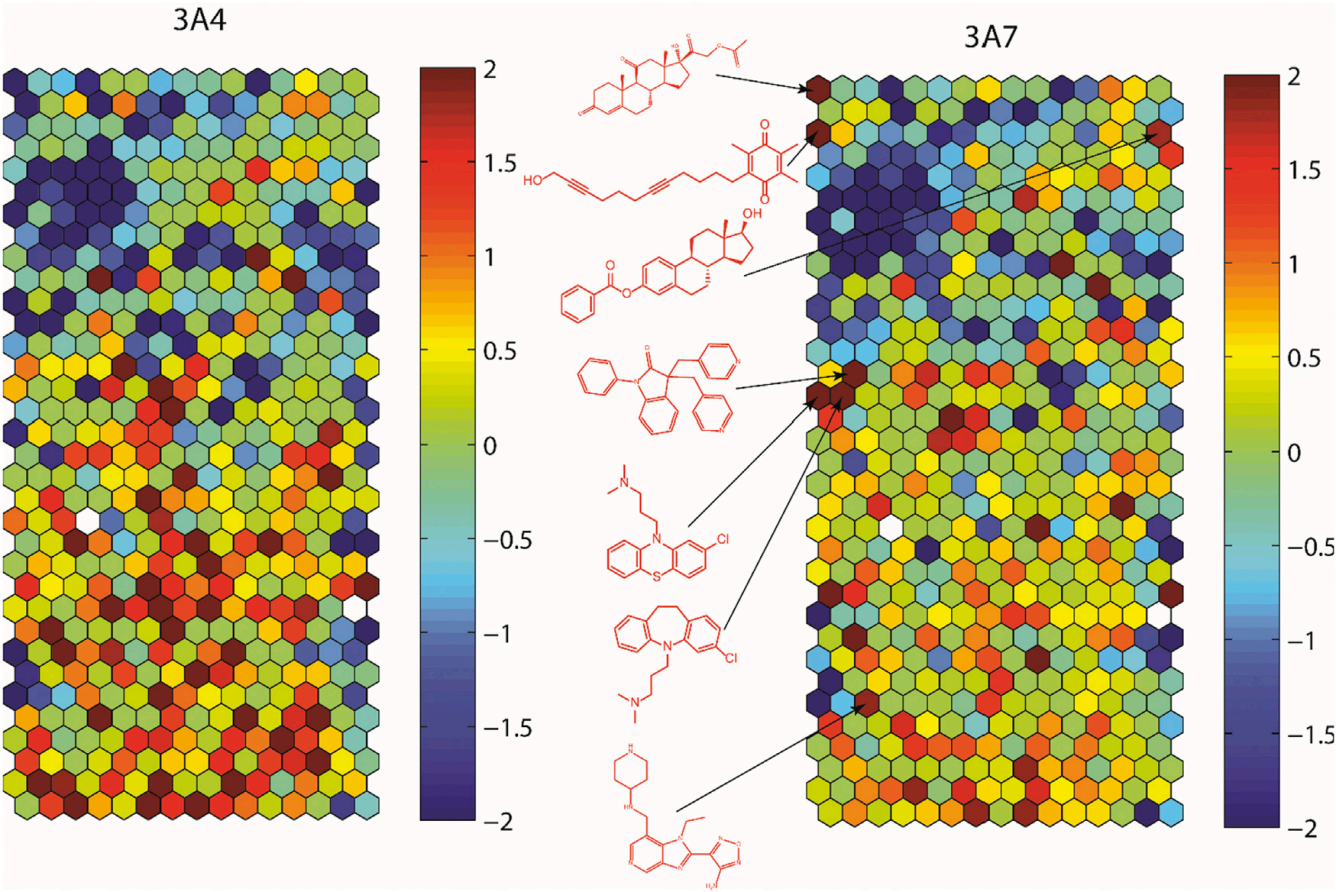
Results of structure clustering of activity of compounds screened in CYP3A4 or CYP3A7. In the heat maps, each hexagon represents a cluster of compounds with structural similarity. Red colored clusters represent structures enriched with active compounds and blue colored clusters represent structures with minimal active compounds. Coloring is scaled by the negative log10 of the *p*-values. Compound structures show the examples of known drug groups active against CYP3A4 or CYP3A7 supersomes.

To assess structural features associated with CYP3A7 or CYP3A4 activity, the entire 5K compounds were clustered based on structural similarity (Figure 3). Each cluster was analyzed for enrichment towards CYP3A4 (left panel of Figure 3) and CYP3A7 (right panel of Figure 3) activity. In Figure 3, each hexagon represents a cluster of structurally similar compounds, with color representing the *p-*values calculated using the Fisher’s exact test. Clusters enriched with active compounds are colored in red, whereas blue clusters represent an underrepresentation of active compounds compared to the library average. Figure 3 shows that compounds of different structural classes were active against CYP3A7 compared to CYP3A4 as indicated by the different red/blue color distributions in the CYP3A7 heat map *vs.* the CYP3A4 heat map. For example, the clusters labeled with an example compound structure in Figure 3 are enriched with CYP3A7 active compounds (right panel; colored in dark red) and are deficient of CYP3A4 active compounds (hexagons in the same locations in the left panel are colored in green or blue). The raw data for Figure 3 can be found in Supplementary Material S1.

The average physicochemical properties of CYP3A7 and CYP3A4-selective hits or the common hits were further evaluated using the ADMET Predictor™ software (Table 1). To better understand if there are marked characteristics that make a compound CYP3A7-selective compared to the compounds that were CYP3A4 selective in this data set, their molecular descriptors were compared. The SlogP was one log unit higher in CYP3A4-selective than CYP3A7-selective hits (common CYP3A4/3A7 inhibitors excluded) (*p* < 0.001), while CYP3A7 hits have a lower molecular weight and topological polar surface area (Table 1). CYP3A7-selective hits also had lower number of hydrogen bond acceptor and donors than CYP3A4-selective hits. CYP3A7 hits also had lower numbers of aromatic rings and fraction of aromatic bonds (*p* < 0.001). The frequency of the descriptor for steroid-like fused ring subunit was determined to be 10 times higher in CYP3A7 hits than CYP3A4 hits (*p* < 0.001).

**TABLE 1:** Comparison of molecular descriptors between common (both CYP3A4 and-CYP3A7), CYP3A4-selective and CYP3A7-selective hits. Data shown as mean ± SD.

Molecular descriptors	Common (CYP3A4–CYP3A7) *n* = 2,216	CYP3A4 *n* = 573	CYP3A7 *n* = 782	*p*-value
SlogP	4 ± 2	4 ± 2	3 ± 2	<0.001
Molecular weight	406 ± 117	411 ± 137	355 ± 102	<0.001
Topological polar surface area	83 ± 41	83 ± 45	76 ± 41	<0.001
Hydrogen bond donor	2 ± 1	2 ± 2	1 ± 1	0.08
Hydrogen bond acceptor	4 ± 3	5 ± 3	4 ± 2	<0.001
Fraction of aromatic bonds	0.5 ± 0.2	0.5 ± 0.2	0.4 ± 0.2	<0.001
Number of aromatic rings	3 ± 1	3 ± 1	2 ± 1	<0.001
Indicator variable for steroid like fused ring subunit	0.002 (4 cpds)	0.002 (1 cpd)	0.012 (9 cpds)	<0.001

*p* value between CYP3A4-selective and CYP3A7-selective hits; common CYP3A4 and CYP3A7 not included. cpd = compounds.

### Identification of CYP3A7-Selective Hits From Quantitative High-Throughput Screening

**FIGURE 4:**
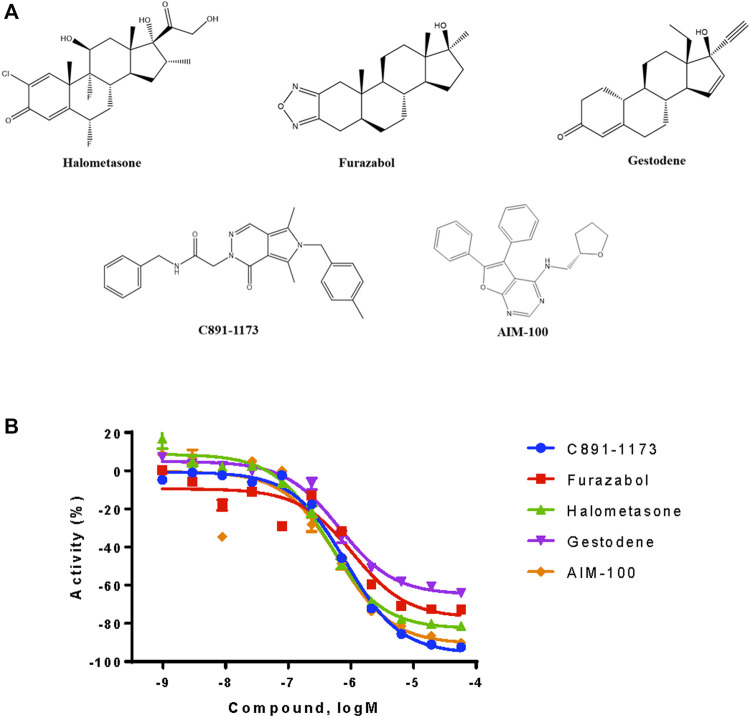
Structures and inhibition profile of CYP3A7 inhibitors based on P450-Glo CYP3A7 assay with a >10-fold IC_50_ difference against CYP3A4.

From the qHTS assay results, several CYP3A7-selective inhibitors were identified based on potency ≤1 μM, efficacy ≥65% and a ≥10-fold IC_50_ difference with CYP3A4. To confirm the finding, 383 CYP3A7-selective and 245 CYP3A4-selective compounds were re-tested. The confirmation rate for CYP3A7 was 86% and confirmation rate for CYP3A4 was 92%. As shown in Table 2, halometasone, AIM-100, gestodene, C891-1,173, and furazabol inhibited the activity of CYP3A7 with an IC_50_ of 0.43, 0.45, 0.61, 0.77, and 0.97 μM, respectively (Table 2; Figure 4). These compounds were all at least ten times less potent in the CYP3A4 qHTS assay.

**TABLE 2:** The activity and maximum response of inhibitors were compared between CYP3A4 and CYP3A7. Data shown as mean ± SD.

Compounds	CYP3A7 IC_50_ (µM)	CYP3A7 efficacy (%)	CYP3A4 IC_50_ (µM)	CYP3A4 efficacy (%)
Halometasone	0.43 ± 0.05	−91 ± 3	14.9 ± 2.54	−71 ± 8
AIM-100	0.45 ± 0.06	−98 ± 4	17.2 ± 0.01	−65 ± 2
Gestodene	0.61 ± 0.07	−65 ± 1	13.1 ± 3.88	−71 ± 5
C891-1,173	0.77 ± 0.01	−89 ± 1	15.3 ± 0.01	−48 ± 5
Furazabol	0.97 ± 0.01	−68 ± 1	19.3 ± 0.01	−42 ± 1

### Halometasone Docking in CYP3A4 and CYP3A7 Model

**FIGURE 5:**
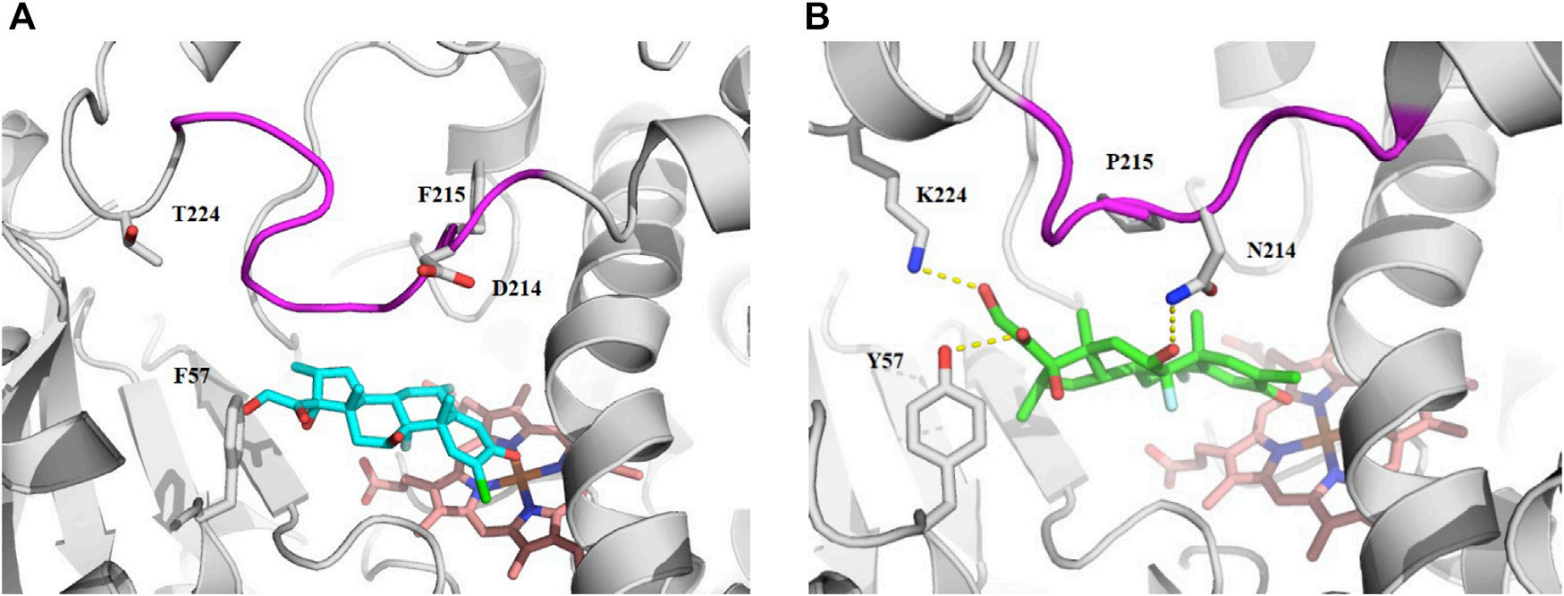
The predicted binding mode of halometasone in the active cavity of CYP3A4 **(A)** and CYP3A7 **(B)**. Key residues interacting with the bound substrate are shown as sticks and H-bonding interactions are shown with dotted line. The flexible FG loop is shown in magenta and the heme group is shown in orange.

Since the physicochemical descriptors and structure cluster analysis indicated sterol-based compounds are more CYP3A7-selective, we next investigated a docking simulation of the most potent CYP3A7 inhibitor, halometasone. Halometasone was docked into the active site of CYP3A4 *via* interactions with heme and lipophilic interactions with the PHE-clusters. A similar binding model of halometasone was predicted within the active site of CYP3A7 *via* interactions with heme and the FG loophydrophobic interactions, which is generally conserved in both protein targets (Torimoto et al., 2007; Kandel et al., 2017). Interestingly, halometasone was found to interact with the FG loop of CYP3A7 closely by forming a H-bond with ASN214, as well as two additional H-bonds with Y57 and K227 surrounding the active site. These H-bond interactions were not observed in the binding model of CYP3A4 due to the different residues on these positions (Figure 5). The predicted binding energies of halometasone with CYP3A4 and CYP3A7 were −7.85 and −8.15 kcal/mol, respectively, suggesting that halometasone binds to CYP3A7 with a higher affinity than CYP3A4. Analysis of the structural binding models showed that the FG loop at the catalytic site of CYP3A7 is packed in a more closed form as compared to the loop in CYP3A4. This conformational change may be due partly to the residues ASP214 and Phe215 in CYP3A4 changing to ASN214 and Pro215 in CYP3A7 (Figure 5B). Consequently, binding of halometasone with CYP3A7 may induce a conformational change of the catalytic loop in such a manner that it interacts with the small molecule more tightly and locks it in an inactive form, whereas halometasone adopts an active and open conformation in the active site of CYP3A4 allowing for catalysis. This may explain why halometasone inhibits CYP3A7 whereas it is metabolized by CYP3A4.

### Identification of CYP3A7-Selective Substrates From Metabolic Stability Assay

**FIGURE 6:**
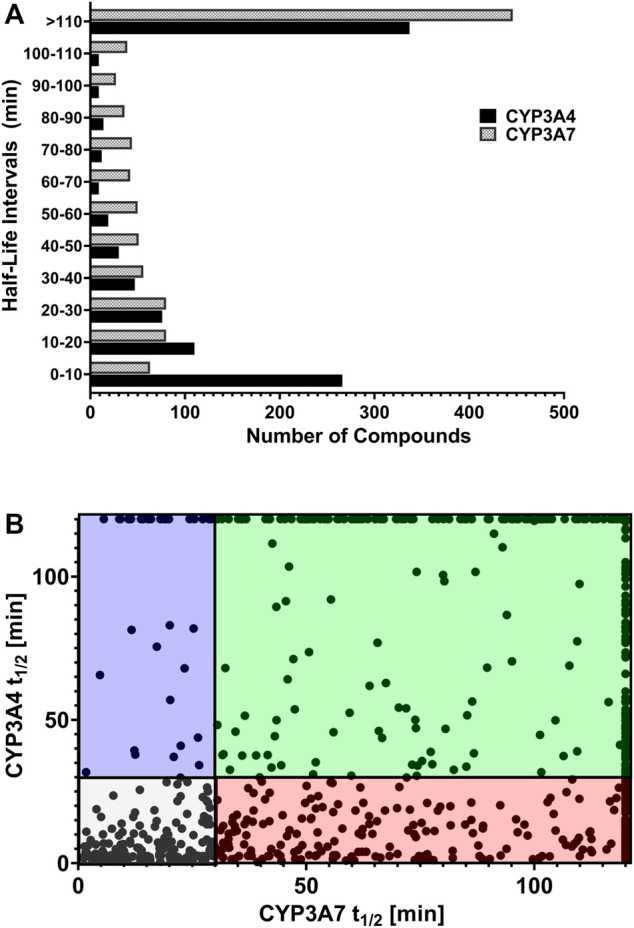
**(A)** Distribution of substrates against CYP3A4 and CYP3A7 based on half-life obtained in substrate depletion assay. **(B)** Correlation of t_1/2_ between substrates in CYP3A4 and CYP3A7 substrate depletion assays. Blue box: CYP3A7 preferred substrates (t_1/2_ ≤ 30 min for CYP3A7 and >30 min for CYP3A4). The CYP3A7-selective substrates are shown at the top boundary line (t_1/2_ ≤ 30 min for CYP3A7 and >120 min for CYP3A4; see details in text). Red box: CYP3A4 preferred substrates (t_1/2_ ≤ 30 min for CYP3A4 and >30 min for CYP3A7). Green box: Weak substrates (t_1/2_ > 30 min for both enzymes). Gray box: Common substrates (t_1/2_ < 30 min for both enzymes).

We reasoned that CYP3A7 substrates and inhibitors would not be distinguishable from each other in the qHTS P450-Glo assay since both could exhibit an inhibitory profile using this assay format. To characterize hits as substrates versus competitive inhibitors, we performed a high-throughput metabolic stability assay using 1,000 randomly selected compounds from the qHTS library. Compounds were classified as selective CYP3A7-substrates if they had a half-life ≤30 min in CYP3A7 supersomes and >120 min in CYP3A4 supersomes, and the inverse criteria were applied to identify CYP3A4 substrates. Out of 1,120 compounds tested, mass spectrometry (MS) data were successfully obtained for 1,015 compounds. From this set, 223 compounds (22%). 223/1015=0.219 were categorized as CYP3A7-substrates and 452 compounds (45%) as CYP3A4-substrates (t_1/2_ < 30 min) as shown in Figure 6A, which shows the distribution of half-life (t_1/2_) of compounds for the two enzymes in intervals of 10 min. Among the CYP3A7-substrates list, 159 compounds overlapped with CYP3A4-substrates (71% of overall CYP3A7 substrates) as shown in Figure 6B. Out of the remaining 64 compounds, 28 compounds did not show data for CYP3A4 but 36 compounds (Blue box in Figure 6B) were found to be preferred CYP3A7 substrates (t_1/2_ ≤ 30 min for CYP3A7 and >30 min for CYP3A4). These compounds were re-examined in the metabolic stability assay which confirmed the compound’s behavior (data not shown). Among these 36 compounds, 22 compounds were identified as CYP3A7-selective compounds (i.e., t_1/2_ ≤ 30 min for CYP3A7 and >120 min for CYP3A4; Table 3). Moreover, based on the 30 min threshold, 293 compounds were found to be selective substrates of CYP3A4, which represents 65% of CYP3A4 substrates.

**TABLE 3:** List of CYP3A7-selective substrates compared to CYP3A4 based on t_1/2_ in supersomes.

Compound name	3A7 t_1/2_ (min)	3A4 t_1/2_ (min)	Pharmacological activity	Metabolism route/Enzyme involved	References
Asparagine Monohydrate	5.6	>120	Dietary supplement	Hydrolyzed by Asparaginase	
Fenoldopam	9.0	>120	Antihypertensive	Sulfation, Methylation, and Glucuronidation. No CYP450 involvement	Klecker and Collins (1997)
Eucalyptol	9.2	>120	Cough supressant	3A4, 3A5 Km > 12 for all metabolites	Duisken et al. (2005)
Etofylline	11.0	>120	Vasodilation and relaxation of smooth muscle	Not Reported	
DB-07268	11.6	>120	Diabetes	Not Reported	
Famciclovir	13.8	>120	Antiviral (Herpes)	Aldehyde Oxidase	Rashidi et al. (1997)
Glutamine	14.3	>120	Dietary supplement	Glutamine synthetase and phosphate-dependent glutaminase	Cruzat et al. (2018)
Todralazine	15.4	>120	Antihypertensive	Not Reported	
Clevudine	15.4	>120	Antiviral (Hepatitis B)	Phosphorylation	Niu et al. (2008)
Cotinine	15.9	>120	Antidepressant, plant metabolite	CYP2A13	Bao et al. (2005)
Idasanutlin	18.0	>120	Antineoplastic	CYP3A4/2C8	Umehara et al. (2022)
AZD-1283	18.1	>120	Antiplatelet Agent (trombosis)	Not Reported	
Tylosin	18.2	>120	Antibiotic	CYP1A1	Bart et al. (2020)
MK-8245	18.9	>120	Type 2 Diabetes Mellitus, dyslipidemia	Not Reported	
Piconol	19.4	>120	Associated with ibuprofen, antiinflamatory	Not Reported	
TC-G 1006	19.9	>120	Immunossupressive	Not Reported	
Nicoboxil	24.4	>120	Analgesic	Esterases	Drug Bank: DB12911
Tandutinib	24.5	>120	Antineoplastic	CYP mediated	Al-Shakliah et al. (2021)
Levocarnitine	25.5	>120	Dietary supplement, carnitine deficiency	Bacterial microflora	Pekala et al. (2011)
Alisertib	27.3	>120	Antineoplastic	CYP3A4, Acyl Glucuronidation	Zhou et al. (2018)
AP-768	28.7	>120	Eosinophilic asthma	Not Reported	
Iopromide	29.2	>120	X-ray contrast	Not Reported	

## Discussion

CYP3A7 is the primary CYP3A enzyme present in neonates, while CYP3A4 is predominant in adults (Stevens et al., 2003; Zane et al., 2018). CYP3A7 expression can also be found in a subset of adult populations (Sim et al., 2005; Smit et al., 2005) and is highly expressed in the endometrium of women during the follicular phase of pregnancy (Sarkar et al., 2003). There is lack of data regarding CYP3A7-mediated metabolism and inhibition compared to CYP3A4. Therefore, our aim was to evaluate the difference between CYP3A7 and CYP3A4 substrates and inhibitors.

In this study, we performed the first high-throughput screen of a large collection of 5,000 approved drugs and bioactive compounds for CYP3A7 substrate and inhibitor activity assessment and compared it against CYP3A4. Our assays have shown that slightly more compounds interact (activator or inhibitor) with CYP3A7 over CYP3A4 (Figure 2). This posed us several questions such as does CYP3A7 have ligand promiscuity like CYP3A4 and whether there are marked characteristics of CYP3A7-selective compounds over CYP3A4-selective compounds.

From the P450 Glo assay screening, many compounds from this chemically diverse library (2,216 compounds) inhibited both CYP3A7/CYP3A4 isozymes, while additional 782 compounds were CYP3A7-selective. Further investigation on the characteristics of CYP3A7-selective compounds revealed slightly lower SlogP and topological polar surface area are associated with CYP3A7-selectivity (Table 1). However, it is well established that higher lipophilicity is associated with affinity to CYP3A4, which might facilitate H-bond interactions with phenylalanine residues in the active site (Hansch et al., 2004; Sridhar et al., 2012). Our assay data suggest that differences between CYP3A7 and CYP3A4, indicated by significant *p*-values, in parameters such as SlogP, molecular weight, topological polar surface area, hydrogen bond acceptor, fraction of aromatic bonds, and number of aromatic rings, may not be applicable as a general rule of thumb to differentiate CYP3A7 or CYP3A4 selective compounds. However, the number compounds containing steroid-like fused ring subunit showed 10-fold increase to CYP3A7-selective hits and statistically significant *p*-value. This suggests that steroid-like compounds are more likely to be CYP3A7 than CYP3A4 ligands. This information might also help shed light on CYP3A7 biological function during human development after birth and the physiological role it fulfills before maturation of other CYP450s, such as protect the fetus from harmful effects of the mother’s endogenous steroidal hormones (Schuetz et al., 1993).

Some inhibitors identified from this study were reported previously (Kandel et al., 2017; Zane et al., 2018). Godamudunage et al. (2018) showed that azole antifungals inhibited CYP3A4 more potently than CYP3A7. Our structure fragment analysis of CYP3A4 hits also revealed that several azole moieties such as imidazole, pyrazole, and thiazole are statistically significant fragments associated with CYP3A4 inhibition in the qHTS assay. In addition, a steroid-like fused ring backbone is important for CYP3A7 activity (Figure 3; Table 1). We identified several steroid-scaffold containing compounds such as furazabol, and gestodene that had a ≥20-fold higher selectivity for CYP3A7 inhibition (Figure 3). It is known that CYP3A7 is associated with converting DHEA into 16α-DHEA which is then used as the precursor for the estriol biosynthesis in placenta (Leeder et al., 2005). Moreover, CYP3A7 converts testosterone into 2α-hydroxytestosterone in fetal microsomes (Leeder et al., 2005; Kandel et al., 2017). This common theme of steroid-based backbone capable of engaging CYP3A7 activity was also shown by Nakamura et al. (2003) where CYP3A7-mediated carbamazepine 10,11-epoxidation was increased by sulfate-conjugate steroids. Based on previous reports and our experimental data, it can be stipulated that compounds containing steroid-like scaffolds are likely to be CYP3A7-selective ligands.

Since the P450-Glo CYP3A7 and CYP3A4 assays cannot differentiate substrates from competitive inhibitors, it is critical to further dive into the two types of ligands *via* metabolic stability assay. Using the >10-fold IC_50_ difference criteria in the follow-up qHTS assay and adding another parameter of half-life >120 min in CYP3A7 metabolic stability assay, halometasone was identified to be a CYP3A7-competitve inhibitor with an IC_50_ of 430 nM. Also, halometasone was only metabolized by CYP3A4 with a half-life of 22 min but is stable (>120 min) in CYP3A7 metabolic stability assay. Our docking model suggests that halometasone binds stronger with CYP3A7 and forms hydrogen-bonding with the asparagine residue in the FG loops. Previous study by Torimoto et al. (2007) illustrated that difference in amino acid residues in the FG loops are important for CYP3A7 substrate recognition over CYP3A4. Our model showed that upon binding of the ligand, there is a shift in the coordinate system which might render CYP3A7 inactive, while there was no significant change in CYP3A4 conformation. This observation might explain how a sterol-based compound can inhibit CYP3A7 activity instead of being metabolized by this enzyme.

While several CYP3A7-mediated oxidation marker reactions were previously reported with DHEA (Leeder et al., 2005), testosterone (Kandel et al., 2017) and recently with deoxycholate (DCA) (Chen et al., 2019), they are substrates for both CYP3A4 and CYP3A7 (Williams et al., 2002; Leeder et al., 2005; Kandel et al., 2017; Chen et al., 2019). A selective CYP3A7 substrate is yet to be identified, our dataset can be further studied to monitor CYP3A7-selective activity over CYP3A4 in a supersome setting. In our substrate analysis, we performed metabolic stability assay of a 1,000 randomly selected compounds from the 5,000-compound library. Interestingly, CYP3A7 was only able to metabolize 22% of the compounds tested while CYP3A4 metabolized nearly 50% of the compounds (Figure 6). This distinction is important particularly in the setting of fetal and neonatal drug administration, where approved drugs in adults are often administered off-label (Hsieh et al., 2014; Avant et al., 2017). To select potentially selective CYP3A7 substrates, we selected substrates with t_1/2_ less than 30 min for CYP3A7 and more than 120 min for CYP3A4 (to obtain a marked metabolism distinction), and found 22 such compounds (Table 3). These compounds were virtually not metabolized by CYP3A4 during the 60-min incubation but had substantial turnover in the CYP3A7 assay.

Among these compounds, fenoldopam, an antihypertensive drug, is not known to be metabolized by CYP450 (Klecker and Collins, 1997). In this study, we confirm that CYP3A4 does not seem to be involved in its metabolism, however, CYP3A7 t_1/2_ of fenoldopam was only 9 min (Table 3). This information could be influential in understanding fenoldopam disposition in individuals expressing this enzyme significantly, e.g., pregnant woman, fetus, or infants below 1 year old. Idasanutlin, alisertib, and eucalyptol have been reported to be CYP3A4 substrates with very low conversion rates, which corroborates our findings (Duisken et al., 2005; Venkatakrishnan et al., 2015; Umehara et al., 2022). Additionally, we demonstrate that CYP3A7 is involved in their disposition. Another interesting example is famciclovir, whose metabolism is not associated with cytochrome P450 activity but with aldehyde oxidase (Rashidi et al., 1997). However, we show that CYP3A7 rapidly metabolizes famciclovir with a t_1/2_ of 14 min. Apart from these examples, several other compounds can be derived from our dataset which we believe will drive research of CYP3A7 substrates further.

It is important to notice the limitations of the assay when interpreting the data. When comparing qHTS IC_50_ values it is important to notice that there is a difference in substrate and enzyme concentrations. According to the manufacturer protocols, the Promega P450-Glo kit was validated using LUC-BE substrate at 150 µM and CYP3A7 at final concentration of 20 nM. However, for CYP3A4 the validated method utilizes Luc-PPXE at 15 μM and 10 nM enzyme concentration.

In our experimental approach, the differences in protein binding in the two systems and differences in marker substrates were not considered when calculating IC_50_ values. This may be one factor contributing to the disconnection between qHTS and metabolic stability data, where both enzymes were tested at 27.3 nM. On metabolic stability testing, we have not performed minus-NADPH controls; therefore, common substrates, e.g., with considerable depletion for both enzymes, may have the influence of endogenous insect cell components, CYP450 co-factors, chemical degradation or compound precipitation. Moreover, in this work, we aimed to study compound specificities against CYP3A4 and CYP3A7; however, CYP3A5 may be a confounding factor in drug metabolism. We plan to pursue CYP3A5 evaluation as part of a follow up study.

In summary, a large collection of compounds was tested for their activity against CYP3A7, for comparison with the respective CYP3A4 dataset. Contrary to current understanding, our P450-Glo assay revealed that CYP3A7 inhibition showed greater variety of ligands compared to CYP3A4, indicative of ligand promiscuity (Gorski et al., 1994; Ohmori et al., 1998; Williams et al., 2002; Nakamura et al., 2003). However, this does not translate in the metabolic stability assay, where only a small subset of compounds were capable of being metabolized by CYP3A7. This difference in metabolic properties is important in the NICU setting where approved drugs in adults are administered to neonates. Our structural and fragmentation analysis further showed that sterol-based compounds could act as inhibitors of CYP3A7, implicating possible connections with the role of CYP3A7 during physiological development, which remains at large. Our dataset sheds light to several new potential CYP3A7-selective inhibitors and substrates. The large high-quality dataset generated from this study can be used for future CYP3A7 studies including developing quantitative structure-activity relationship (QSAR) model or ligand-based drug design.

## Data Availability

The original contributions presented in the study are included in the article/Supplementary Material, further inquiries can be directed to the corresponding authors.
